# Development and validation of a clinical prediction model for pneumonia - associated bloodstream infections

**DOI:** 10.3389/fcimb.2025.1531732

**Published:** 2025-03-18

**Authors:** Zhitong Zhou, Shangshu Liu, Fangzhou Qu, Yuanhui Wei, Manya Song, Xizhou Guan

**Affiliations:** ^1^ The Graduate School, Liberation Army Medical College, Beijing, China; ^2^ Department of Cardiology, Shaanxi Provincial People’s Hospital, Xian, Shanxi, China; ^3^ School of Medicine, Nankai University, Tianjin, China; ^4^ Department of Pulmonary and Critical Care Medicine, Liaocheng People’s Hospital, Liaocheng, Shandong, China; ^5^ Department of Pulmonary and Critical Care Medicine, The Eighth Medical Centre, Chinese People's Liberation Army (PLA) General Hospital, Beijing, China

**Keywords:** pneumonia, bloodstream infections, bacteremia, risk factor, early diagnosis, prediction model

## Abstract

**Purpose:**

The aim of this study was to develop a valuable clinical prediction model for pneumonia-associated bloodstream infections (PABSIs).

**Patients and methods:**

The study retrospectively collected clinical data of pneumonia patients at the First Medical Centre of the Chinese People’s Liberation Army General Hospital from 2019 to 2024. Patients who met the definition of pneumonia-associated bloodstream infections (PABSIs) were selected as the main research subjects. A prediction model for the probability of bloodstream infections (BSIs) in pneumonia patients was constructed using a combination of LASSO regression and logistic regression. The performance of the model was verified using several indicators, including receiver operating characteristic (ROC) curve, calibration curve, decision curve analysis (DCA) and cross validation.

**Results:**

A total of 423 patients with confirmed pneumonia were included in the study, in accordance with the Inclusion Criteria and Exclusion Criteria. Of the patients included in the study, 73 developed a related bloodstream infection (BSI). A prediction model was constructed based on six predictors: long-term antibiotic use, invasive mechanical ventilation, glucocorticoids, urinary catheterization, vasoactive drugs, and central venous catheter placement. The areas under the curve (AUC) of the training set and validation set were 0.83 and 0.80, respectively, and the calibration curve demonstrated satisfactory agreement between the two.

**Conclusion:**

This study has successfully constructed a prediction model for bloodstream infections associated with pneumonia cases, which has good stability and predictability and can help guide clinical work.

## 1 Introduction

Pneumonia is a prevalent acute respiratory infection that primarily affects the alveoli and distal bronchial tree. Pneumonia is classified according to the source of infection, with the distinction between community-acquired pneumonia (CAP) and hospital-acquired pneumonia (HAP). The latter category includes ventilator-associated pneumonia (VAP) (Torres et al., 2021). The most prevalent clinical manifestations of the disease include cough, dyspnea, chest pain, sputum production, and fatigue (Metlay et al., 1997; Lamping et al., 2002). Pneumonia represents a significant public health concern, with high morbidity rates observed across all age groups and a strong association with both short- and long-term mortality. The 2019 Global Burden of Disease (GBD) study revealed that approximately 489 million individuals worldwide had been affected by lower respiratory tract infections (LRTIs), encompassing conditions such as pneumonia and bronchiolitis. The demographic groups most affected by these infections were children below five years of age and adults above 70 years of age (Abbafati et al., 2020). Bloodstream infections (BSIs) are serious systemic infections that can readily lead to sepsis and multiple organ dysfunction syndrome (MODS), and have a high mortality rate. Consequently, bloodstream infections have become a significant challenge to public health globally (Kern and Rieg, 2020). Pneumonia represents the most common cause of BSIs, accounting for approximately half of all cases (Angus and van der Poll, 2013). In the event that patients diagnosed with pneumonia fail to receive timely or appropriate treatment, they may develop bloodstream infections or even sepsis as their condition progresses. The incidence of bloodstream infections in modern respiratory wards has been increasing year on year, and this may be related to a number of factors, including an ageing population, increased use of immunosuppressants and rising antibiotic resistance. Severe complications of pneumonia, such as bacteremia and sepsis, represent a significant threat to patient survival.

The progressive advancement of data measurement technology and analytical methods is facilitating a gradual transition in the field of medicine from the era of evidence-based medicine to the era of precision medicine. As a consequence of this transition, the role of clinical prediction models is becoming increasingly significant within the field of healthcare, thereby facilitating the development of more sophisticated and tailored healthcare services. Logistic regression, a widely employed statistical technique, plays a pivotal role in the development of clinical prediction models. However, due to the relatively small sample sizes of some studies, problems such as overfitting are common in model construction, which limits the generalizability and practical application performance of the model. To address this challenge, LASSO regression (Least Absolute Shrinkage and Selection Operator) is an effective strategy that introduces a penalty term to reduce the number of variables in the model, thereby enhancing the stability, interpretability and predictive performance of the model. Consequently, an increasing number of researchers are employing LASSO in conjunction with logistic regression to develop more precise and dependable clinical prediction models.

Currently, there is a dearth of clinical research on bloodstream infections in patients with pneumonia, and there is no consensus on the definition of pneumonia-associated bloodstream infections. In light of these considerations, this study has been designed with the objective of addressing this clinical challenge with a view to preventing pneumonia patients from developing bloodstream infections, thereby avoiding the deterioration of their condition to sepsis. The ultimate objective is a reduction in mortality. The objective of this study is to facilitate an early diagnosis and timely treatment of pneumonia-associated bloodstream infections, with the aim of improving outcomes.

## 2 Patients and methods

### 2.1 Study subjects

This study employed a retrospective research design to collate data on inpatients diagnosed with pneumonia at the PLA General Hospital’s First Medical Centre between May 2019 and May 2024.

The diagnostic criteria for pneumonia-associated bloodstream infections were as follows: (1) the patient presented with pneumonia as the initial symptom and a definitive diagnosis was reached; (2) the results of sputum and blood cultures were consistent with the same pathogen; and (3) all other potential sources of infection were excluded.

### 2.2 Inclusion criteria

The following inclusion criteria were employed:

1. Age > 18 years;
2. Pneumonia-associated clinical manifestations (any one of the following four manifestations is sufficient): (1) A recent cough with sputum or worsening of pre-existing respiratory symptoms, with or without purulent sputum, chest pain, dyspnea, and hemoptysis; (2) Fever; (3) Evidence of lung consolidation (voice raised, percussion dull) and/or wet rales; (4) Peripheral blood leukocyte count >10×10^9^/L or <4×10^9^/L, with or without nuclear shift to the left;
3. Chest imaging demonstrates the presence of new patchy infiltrates, lobular or segmental consolidation, ground-glass opacities, or interstitial changes, with or without pleural effusion;
4. Sputum and blood samples are obtained for culture.

### 2.3 Exclusion criteria

The exclusion criteria were as follows:

1. The patient was below the age of 18 years;
2. The diagnosis of pulmonary infection was uncertain;
3. Sputum and blood culture specimens were not obtained.

### 2.4 Study variables

The study collected patients’ personal information and clinical data by searching the hospital’s electronic medical record system. All baseline characteristics were recorded within a 24-hour period.

Data were collected from each patient for statistical analysis, including:(1) Demographic characteristics: sex, age, body mass index (BMI), date and time of admission; (2) Vital signs: body temperature, heart rate, respiratory rate, blood pressure (systolic and diastolic), and mean arterial pressure (MAP); (3) Personal lifestyle history: smoking and alcohol consumption; (4) History of chronic disease: Hypertension, Diabetes, coronary heart disease (CAD), Congestive Heart Failure, Atrial Fibrillation (AF), Arrhythmia, Hyperlipidemia, Cerebral Infarction, Chronic Respiratory Disease, Alzheimer’s Disease, Parkinson’s Disease, Thyroid Dysfunction, Renal Insufficiency, Benign Prostatic Hyperplasia (BPH), Gastrointestinal Bleeding, Chronic Gastritis, Reflux Esophagitis, Fatty Liver, Cirrhosis, Malignant Tumors, Hematologic Diseases, Lymphoma, Rheumatic Diseases of Immune System; (5) Surgical and treatment history: major surgery(Refers to surgery involving major organs or systems that carries a higher risk, longer recovery time, and may have a significant impact on the patient’s quality of life. This includes, but is not limited to: heart surgery, brain surgery, organ removal, etc.), recent surgery(Refers to surgery that the patient has had within the last 2 weeks), long-term antibiotic treatment(Refers to the use of antibiotics for ≥2 weeks and ≥1 type), radiotherapy, chemotherapy, hemodialysis and organ transplant history; (6) Use of medical devices: nasogastric tube, urinary catheter, central venous catheter placement and drainage tube; (7) Implementation of nutritional support and therapeutic measures: Total parenteral nutrition, invasive mechanical ventilation, sedatives, anticoagulants, vasopressors and glucocorticoids; steroids; (8) Laboratory Indicators: Sputum and blood microbial culture, pH, PaO2, PaCO2, oxygenation index, bicarbonate, lactate, white blood cell count, neutrophil percentage, lymphocyte percentage, hemoglobin, platelet count, C-reactive protein (CRP), interleukin-6 (IL-6), procalcitonin (PCT), serum albumin, total serum albumin, alanine aminotransferase (ALT), aspartate aminotransferase (AST), total bilirubin, direct bilirubin, urea, serum creatinine, serum potassium concentration, serum sodium ion concentration, serum chloride ion concentration, prothrombin time (PT), activated partial thromboplastin time (APTT), prothrombin time activity (PTA), fibrinogen, D-dimer.

### 2.5 Missing data handling

To address the issue of missing data during the data processing stage, the following measures were implemented. In cases where more than 50% of the data was missing, the decision was taken to delete the entire data set. Secondly, if the proportion of missing values for a specific variable exceeded 21%, the variable was excluded from the analysis. In cases where missing values were present for variables within the remaining case data, multiple imputation techniques were employed to ensure the integrity and accuracy of the data analysis.

### 2.6 Sample size estimation

This study is a single-center, retrospective cohort design aimed at developing a multifactorial regression model to predict pneumonia-associated bloodstream infections (PABSI). The study population was selected based on the criterion of concordance between sputum and blood culture results. Given the limitations of clinical resources, sample size estimation was conducted with practical feasibility in mind, ensuring that the statistical requirements were met.

#### 2.6.1 Requirements for multifactor regression analysis (Peduzzi guidelines)

A maximum of six outcome-related predictor variables were intended to be included in the model to balance explanatory power and predictive accuracy, while maintaining simplicity. According to the Peduzzi guideline, each predictor variable should correspond to at least 10 outcome events (EPV ≥ 10) to prevent overfitting and ensure the stability and accuracy of model estimates. Therefore, with six predictor variables, a minimum of 60 outcome events (6 × 10 = 60) is required.

#### 2.6.2 Calculation formula and process

**Figure 1:**
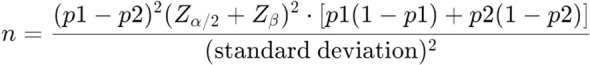
Sample size calculation formula.

To ensure adequate statistical power, sample size calculations were conducted as follows: assuming the incidence of bloodstream infection (BSI) in the exposed group (p_1_) is 20% and in the unexposed group (p_2_) is 5%, with a Type I error rate (α) set at 0.05 and a power (1 - β) of 80%. The sample size was calculated using the formula (**Figure 1**):

where Z_α/2_ is the critical value for the standard normal distribution (1.96 for α = 0.05), and Z_β_ is the critical value for statistical power (0.84 for 80% power). The sample size was calculated as n = 84 to ensure the study would meet the statistical requirements.

Based on the above calculations, the minimum sample size required for this study was 146 cases, with 73 cases in each of the exposed and unexposed groups, to achieve 80% power.

### 2.7 Statistical processing

The statistical analysis was conducted using SPSS 26.0 and R 4.4.1. Quantitative data are expressed as mean ± standard deviation, and for non-normally distributed data, the median and interquartile range are employed. Categorical data are displayed as frequencies or percentages. In order to conduct a comparative analysis of continuous variables, the t-test or Mann-Whitney U test was employed. Similarly, the chi-square test or Fisher’s exact test was used for the comparison of categorical variables. The data was randomly divided into a training set and a validation set in a 1:1 ratio.

In the feature selection phase, a combination of LASSO regression and logistic regression was employed. Initially, all data were subjected to univariate logistic regression analysis to identify potentially pertinent variables with a P-value less than 0.05. Subsequently, LASSO regression was performed, and the 1 standard error (1se) rule was employed to ascertain the optimal penalty coefficient and exclude non-representative variables. Subsequently, a further multivariate logistic regression analysis was conducted to evaluate the independent effects of these variables on the outcome, with the corresponding odds ratios (OR) and 95% confidence intervals (CI) subsequently calculated.

Once the regression equation had been established, a line chart was employed to represent the prediction model. An ROC curve was constructed for the purpose of evaluating the model’s performance, and the AUC value was calculated for the purpose of evaluating its diagnostic ability. The Hosmer-Lemeshow test was employed to evaluate the model’s goodness of fit, while a calibration curve was plotted to assess the calibration effect of the model. Moreover, decision curve analysis (DCA) was employed to determine the clinical utility of the model. In this study, a statistically significant level was set at P < 0.05.

## 3 Results

### 3.1 Patient demographics

A total of 423 patients were enrolled in this study, including 211 patients in the training group and 212 patients in the test group. This sample size significantly exceeded the calculated minimum requirement, thereby ensuring the statistical validity of the study. All patient data were complete and suitable for analysis.

A comparison of the baseline data from the two cohorts revealed significant differences between the two groups with respect to partial pressure of arterial carbon dioxide (Paco_2_; P = 0.036), lymphocyte percentage (P = 0.040), history of diabetes (P = 0.008), and use of drainage tubes (P = 0.025). No significant differences were identified for the remaining indicators (all p-values were greater than 0.05). For further details, please refer to **Table 1**.

**Table 1:** Clinical characteristics and demographic of two groups.

Variables	Total (n = 423)	test (n = 212)	train (n = 211)	*P*
Age/year	79.00 (67.00, 87.00)	78.00 (64.75, 87.00)	80.00 (69.00, 87.00)	0.214
BMI	22.90 (20.85, 24.80)	23.19 (21.08, 25.00)	22.60 (20.35, 24.55)	0.101
Temperature/°C	36.60 (36.40, 36.80)	36.50 (36.40, 36.80)	36.60 (36.40, 36.80)	0.299
Heartbeat/BPM	82.00 (76.00, 93.00)	83.00 (77.75, 93.25)	82.00 (75.00, 90.50)	0.116
Respiratory rate/BPM	20.00 (19.00, 20.00)	20.00 (19.00, 20.00)	20.00 (19.00, 20.00)	0.957
Systolic pressure/mmHg,	128.00 (120.00, 139.00)	128.00 (119.75, 140.00)	128.00 (120.00, 139.00)	0.942
Diastolic pressure/mmHg	73.00 (65.00, 81.00)	73.00 (65.00, 81.00)	73.00 (66.00, 81.00)	0.473
MAP	91.67 (84.67, 99.83)	92.00 (84.00, 99.42)	91.33 (85.33, 100.67)	0.669
PH	7.43 (7.39, 7.47)	7.43 (7.39, 7.47)	7.43 (7.40, 7.47)	0.804
PaO_2_/mmHg,	85.58 (73.90, 94.36)	85.75 (76.62, 95.55)	85.20 (71.00, 93.50)	0.196
PaCo_2_/mmHg	37.28 (33.05, 40.16)	37.05 (32.93, 39.32)	37.79 (33.95, 40.80)	0.036
Oxygenation index	264.00 (227.00, 297.00)	264.86 (233.00, 297.50)	263.00 (221.00, 290.50)	0.217
HCO^3-^/(mmol/L)	24.60 (23.20, 26.39)	24.50 (23.20, 26.05)	24.70 (23.18, 26.70)	0.414
Lac/(mmol/L)	1.56 (1.10, 2.08)	1.64 (1.10, 2.07)	1.42 (1.00, 2.09)	0.253
WBC/(10^9^/L)	7.47 (5.55, 10.49)	7.42 (5.55, 10.52)	7.54 (5.56, 10.41)	0.993
N/%	0.76 (0.63, 0.85)	0.75 (0.62, 0.84)	0.77 (0.66, 0.86)	0.123
L/%	0.14 (0.08, 0.24)	0.15 (0.09, 0.26)	0.12 (0.07, 0.22)	0.040
Hb/(g/L)	120.00 (106.00, 134.50)	121.00 (107.00, 136.00)	119.00 (104.00, 132.50)	0.245
PLT/(10^9^/L)	202.00 (153.50, 267.00)	204.00 (153.25, 259.00)	200.00 (154.00, 268.50)	0.928
CRP/(mg/dl)	2.31 (0.51, 6.70)	2.33 (0.49, 6.08)	2.25 (0.57, 6.77)	0.770
IL-6/(pg/ml)	27.17 (6.29, 90.51)	30.38 (6.19, 92.52)	24.42 (6.64, 89.66)	0.674
PCT	0.18 (0.07, 1.45)	0.18 (0.08, 1.44)	0.19 (0.07, 1.45)	0.821
Albumin/(g/L)	36.40 (33.10, 39.50)	36.50 (33.30, 39.90)	36.20 (33.00, 39.25)	0.231
Total albumin/(g/L)	67.10 (62.30, 72.40)	67.75 (63.40, 72.50)	66.30 (61.50, 71.90)	0.257
ALT/(U/L)	16.60 (11.20, 26.95)	17.40 (11.70, 27.95)	16.10 (10.55, 25.95)	0.186
AST/(U/L)	20.80 (16.00, 29.15)	20.60 (16.50, 29.07)	21.00 (15.50, 29.15)	0.482
Total bilirubin/(umol/L)	8.50 (5.70, 12.20)	8.40 (5.70, 12.12)	8.60 (5.80, 12.30)	0.573
Direct bilirubin/(umol/L)	3.30 (2.20, 5.20)	3.20 (2.20, 5.00)	3.30 (2.30, 5.40)	0.478
Urea/(mmol/L)	5.65 (4.27, 7.97)	5.42 (4.24, 7.50)	5.87 (4.29, 8.38)	0.326
Creatinine/(umol/L)	74.20 (59.80, 91.20)	75.05 (61.25, 91.32)	73.00 (58.95, 89.60)	0.451
K/(mmol/L)	3.78 (3.49, 4.20)	3.78 (3.48, 4.17)	3.79 (3.49, 4.22)	0.556
Na/(mmol/L)	136.80 (132.95, 140.00)	136.60 (133.20, 139.72)	136.90 (132.85, 140.30)	0.510
Cl/(mmol/L)	100.70 (96.90, 104.25)	100.60 (96.55, 103.62)	101.10 (97.05, 104.85)	0.306
PT/s	13.70 (12.90, 14.70)	13.60 (12.80, 14.62)	13.70 (13.00, 14.75)	0.233
APTT/s	36.40 (32.20, 40.70)	36.30 (32.00, 41.23)	36.40 (32.55, 40.00)	0.825
PTA	88.00 (78.00, 98.00)	89.00 (79.07, 99.00)	86.10 (76.00, 97.00)	0.161
Fib	4.52 (3.60, 5.64)	4.55 (3.71, 5.65)	4.49 (3.52, 5.63)	0.674
D-D/(ug/ml)	1.10 (0.52, 2.26)	1.05 (0.50, 2.08)	1.16 (0.55, 2.42)	0.336
Outcome, n (%)				0.151
0	350 (82.74)	181 (85.38)	169 (80.09)	
1	73 (17.26)	31 (14.62)	42 (19.91)	
Sex, n (%)				0.793
1	262 (61.94)	130 (61.32)	132 (62.56)	
2	161 (38.06)	82 (38.68)	79 (37.44)	
Cigarette, n (%)				0.197
0	324 (76.60)	168 (79.25)	156 (73.93)	
1	99 (23.40)	44 (20.75)	55 (26.07)	
Alcohol, n (%)				0.600
0	345 (81.56)	175 (82.55)	170 (80.57)	
1	78 (18.44)	37 (17.45)	41 (19.43)	
Hypertension, n (%)				0.187
0	198 (46.81)	106 (50.00)	92 (43.60)	
1	225 (53.19)	106 (50.00)	119 (56.40)	
Diabetes, n (%)				0.008
0	300 (70.92)	138 (65.09)	162 (76.78)	
1	123 (29.08)	74 (34.91)	49 (23.22)	
Coronary Artery Disease, n (%)				0.718
0	312 (73.76)	158 (74.53)	154 (72.99)	
1	111 (26.24)	54 (25.47)	57 (27.01)	
Heart Failure, n (%)				0.371
0	418 (98.82)	208 (98.11)	210 (99.53)	
1	5 (1.18)	4 (1.89)	1 (0.47)	
Atrial Fibrillation, n (%)				0.235
0	395 (93.38)	201 (94.81)	194 (91.94)	
1	28 (6.62)	11 (5.19)	17 (8.06)	
Arrhythmia, n (%)				0.450
0	416 (98.35)	207 (97.64)	209 (99.05)	
1	7 (1.65)	5 (2.36)	2 (0.95)	
Hyperlipidemia, n (%)				0.092
0	376 (88.89)	183 (86.32)	193 (91.47)	
1	47 (11.11)	29 (13.68)	18 (8.53)	
Cerebral infarction, n (%)				0.683
0	350 (82.74)	177 (83.49)	173 (81.99)	
1	73 (17.26)	35 (16.51)	38 (18.01)	
Chronic respiratory disease, n (%)				0.146
0	367 (86.76)	189 (89.15)	178 (84.36)	
1	56 (13.24)	23 (10.85)	33 (15.64)	
Alzheimer’s disease, n (%)				0.357
0	390 (92.20)	198 (93.40)	192 (91.00)	
1	33 (7.80)	14 (6.60)	19 (9.00)	
Parkinsonism, n (%)				0.425
0	408 (96.45)	206 (97.17)	202 (95.73)	
1	15 (3.55)	6 (2.83)	9 (4.27)	
Gastrointestinal hemorrhage, n (%)				1.000
0	420 (99.29)	210 (99.06)	210 (99.53)	
1	3 (0.71)	2 (0.94)	1 (0.47)	
Chronic gastritis, n (%)				0.812
0	406 (95.98)	203 (95.75)	203 (96.21)	
1	17 (4.02)	9 (4.25)	8 (3.79)	
Gastroesophageal Reflux Disease, n (%)				0.496
0	414 (97.87)	209 (98.58)	205 (97.16)	
1	9 (2.13)	3 (1.42)	6 (2.84)	
Fatty liver, n (%)				1.000
0	421 (99.53)	211 (99.53)	210 (99.53)	
1	2 (0.47)	1 (0.47)	1 (0.47)	
Liver cirrhosis, n (%)				0.997
0	420 (99.29)	211 (99.53)	209 (99.05)	
1	3 (0.71)	1 (0.47)	2 (0.95)	
Malignant tumor, n (%)				0.179
0	355 (83.92)	183 (86.32)	172 (81.52)	
1	68 (16.08)	29 (13.68)	39 (18.48)	
Hematological Diseases, n (%)				0.593
0	409 (96.69)	204 (96.23)	205 (97.16)	
1	14 (3.31)	8 (3.77)	6 (2.84)	
Lymphoma, n (%)				0.994
0	414 (97.87)	208 (98.11)	206 (97.63)	
1	9 (2.13)	4 (1.89)	5 (2.37)	
Rheumatic immune system disease, n (%)				0.552
0	411 (97.16)	207 (97.64)	204 (96.68)	
1	12 (2.84)	5 (2.36)	7 (3.32)	
History of major surgery, n (%)				0.785
0	369 (87.23)	184 (86.79)	185 (87.68)	
1	54 (12.77)	28 (13.21)	26 (12.32)	
Hypothyroidism, n (%)				0.450
0	416 (98.35)	207 (97.64)	209 (99.05)	
1	7 (1.65)	5 (2.36)	2 (0.95)	
Renal insufficiency, n (%)				0.199
0	390 (92.20)	199 (93.87)	191 (90.52)	
1	33 (7.80)	13 (6.13)	20 (9.48)	
Benign Prostatic Hyperplasia, n (%)				0.882
0	382 (90.31)	191 (90.09)	191 (90.52)	
1	41 (9.69)	21 (9.91)	20 (9.48)	
Peripheral Vascular Disease, n (%)				0.436
0	408 (96.45)	203 (95.75)	205 (97.16)	
1	15 (3.55)	9 (4.25)	6 (2.84)	
Long-term antibiotic use, n (%)				0.387
0	401 (94.80)	199 (93.87)	202 (95.73)	
1	22 (5.20)	13 (6.13)	9 (4.27)	
Radiotherapy or chemotherapy, n (%)				0.566
0	392 (92.67)	198 (93.40)	194 (91.94)	
1	31 (7.33)	14 (6.60)	17 (8.06)	
Dialysis, n (%)				0.996
0	418 (98.82)	210 (99.06)	208 (98.58)	
1	5 (1.18)	2 (0.94)	3 (1.42)	
Organ transplant, n (%)				1.000
0	420 (99.29)	210 (99.06)	210 (99.53)	
1	3 (0.71)	2 (0.94)	1 (0.47)	
Recent surgery, n (%)				0.499
0	421 (99.53)	210 (99.06)	211 (100.00)	
1	2 (0.47)	2 (0.94)	0 (0.00)	
Gastric tube, n (%)				0.975
0	299 (70.69)	150 (70.75)	149 (70.62)	
1	124 (29.31)	62 (29.25)	62 (29.38)	
Urinary tube, n (%)				0.709
0	326 (77.07)	165 (77.83)	161 (76.30)	
1	97 (22.93)	47 (22.17)	50 (23.70)	
Central venous catheter, n (%)				0.130
0	332 (78.49)	160 (75.47)	172 (81.52)	
1	91 (21.51)	52 (24.53)	39 (18.48)	
Drainage tube, n (%)				0.025
0	406 (95.98)	208 (98.11)	198 (93.84)	
1	17 (4.02)	4 (1.89)	13 (6.16)	
Total Parenteral Nutrition, n (%)				1.000
0	418 (98.82)	209 (98.58)	209 (99.05)	
1	5 (1.18)	3 (1.42)	2 (0.95)	
Invasive mechanical ventilation, n (%)				0.846
0	398 (94.09)	199 (93.87)	199 (94.31)	
1	25 (5.91)	13 (6.13)	12 (5.69)	
Sedation, n (%)				0.637
0	405 (95.74)	202 (95.28)	203 (96.21)	
1	18 (4.26)	10 (4.72)	8 (3.79)	
Anticoagulant, n (%)				0.329
0	271 (64.07)	131 (61.79)	140 (66.35)	
1	152 (35.93)	81 (38.21)	71 (33.65)	
Vasoactive Drugs, n (%)				0.516
0	383 (90.54)	190 (89.62)	193 (91.47)	
1	40 (9.46)	22 (10.38)	18 (8.53)	
Glucocorticoid, n (%)				0.789
0	270 (63.83)	134 (63.21)	136 (64.45)	
1	153 (36.17)	78 (36.79)	75 (35.55)	

### 3.2 Factor screening

In this study, a univariate logistic regression analysis was initially performed on all included variables, which encompassed demographic characteristics, vital signs, personal history of lifestyle habits, history of chronic diseases, history of surgery and treatment, use of medical equipment, nutrition and supportive care, use of drugs, and laboratory test indicators.

The results of the univariate logistic regression analysis demonstrated that the following variables were significantly associated with pneumonia-associated bloodstream infections (PABSIs) (P < 0.05): coronary heart disease, long-term use of antibiotics, nasogastric tube use, urinary catheter use, and central venous catheter, invasive mechanical ventilation, and sedative drug use, use of vasoactive drugs, use of glucocorticoids, body temperature, heart rate, diastolic blood pressure, and mean arterial pressure (MAP), oxygenation index, urea. For further details, please refer to **Table 2**.

**Table 2:** Univariate logistic regression.

Variables	β	S. E	Z	*P*	OR (95%CI)
Sex					
1					1.00 (Reference)
2	-0.64	0.38	-1.67	0.096	0.53 (0.25 ~ 1.12)
Cigarette					
0					1.00 (Reference)
1	0.30	0.38	0.80	0.421	1.36 (0.65 ~ 2.85)
Alcohol					
0					1.00 (Reference)
1	0.33	0.41	0.80	0.424	1.39 (0.62 ~ 3.13)
Hypertension					
0					1.00 (Reference)
1	0.16	0.35	0.46	0.648	1.17 (0.59 ~ 2.33)
Diabetes					
0					1.00 (Reference)
1	-0.13	0.42	-0.31	0.758	0.88 (0.39 ~ 1.99)
Coronary Artery Disease					
0					1.00 (Reference)
1	0.79	0.36	2.16	0.030	2.19 (1.08 ~ 4.46)
Heart Failure					
0					1.00 (Reference)
1	-13.18	882.74	-0.01	0.988	0.00 (0.00 ~ Inf)
Atrial Fibrillation					
0					1.00 (Reference)
1	-0.16	0.66	-0.24	0.808	0.85 (0.23 ~ 3.11)
Arrhythmia					
0					1.00 (Reference)
1	17.01	1029.12	0.02	0.987	24326759.34 (0.00 ~ Inf)
Hyperlipemia					
0					1.00 (Reference)
1	0.48	0.56	0.87	0.386	1.62 (0.54 ~ 4.83)
Cerebral infarction					
0					1.00 (Reference)
1	0.45	0.42	1.09	0.277	1.57 (0.69 ~ 3.56)
Chronic respiratory disease					
0					1.00 (Reference)
1	0.50	0.44	1.15	0.252	1.65 (0.70 ~ 3.87)
Alzheimer’s disease					
0					1.00 (Reference)
1	-0.31	0.65	-0.47	0.639	0.74 (0.20 ~ 2.65)
Parkinsonism					
0					1.00 (Reference)
1	-0.71	1.07	-0.66	0.508	0.49 (0.06 ~ 4.04)
Gastrointestinal hemorrhage					
0					1.00 (Reference)
1	-13.18	882.74	-0.01	0.988	0.00 (0.00 ~ Inf)
Chronic gastritis					
0					1.00 (Reference)
1	-15.22	848.37	-0.02	0.986	0.00 (0.00 ~ Inf)
Gastroesophageal Reflux Disease					
0					1.00 (Reference)
1	1.45	0.84	1.73	0.083	4.26 (0.83 ~ 21.90)
Fatty liver					
0					1.00 (Reference)
1	15.98	882.74	0.02	0.986	8731034.54 (0.00 ~ Inf)
Liver cirrhosis					
0					1.00 (Reference)
1	17.01	1029.12	0.02	0.987	24326759.33 (0.00 ~ Inf)
Malignant tumor					
0					1.00 (Reference)
1	-0.16	0.46	-0.34	0.735	0.86 (0.35 ~ 2.10)
Hematological Diseases					
0					1.00 (Reference)
1	0.72	0.88	0.82	0.413	2.06 (0.36 ~ 11.66)
Lymphoma					
0					1.00 (Reference)
1	0.01	1.13	0.01	0.996	1.01 (0.11 ~ 9.24)
Rheumatic immune system disease					
0					1.00 (Reference)
1	1.15	0.78	1.47	0.141	3.17 (0.68 ~ 14.76)
History of major surgery					
0					1.00 (Reference)
1	0.68	0.47	1.46	0.144	1.97 (0.79 ~ 4.91)
Hypothyroidism					
0					1.00 (Reference)
1	-14.19	1029.12	-0.01	0.989	0.00 (0.00 ~ Inf)
Renal insufficiency					
0					1.00 (Reference)
1	0.61	0.52	1.17	0.240	1.85 (0.66 ~ 5.13)
Benign Prostatic Hyperplasia					
0					1.00 (Reference)
1	-1.65	1.04	-1.58	0.114	0.19 (0.03 ~ 1.48)
Peripheral Vascular Disease					
0					1.00 (Reference)
1	0.72	0.88	0.82	0.413	2.06 (0.36 ~ 11.66)
Long-term antibiotic use					
0					1.00 (Reference)
1	2.82	0.82	3.42	<.001	16.70 (3.33 ~ 83.81)
Radiotherapy or chemotherapy					
0					1.00 (Reference)
1	0.57	0.56	1.01	0.311	1.77 (0.59 ~ 5.33)
Dialysis					
0					1.00 (Reference)
1	0.71	1.24	0.57	0.565	2.04 (0.18 ~ 23.01)
Organ transplant					
0					1.00 (Reference)
1	15.98	882.74	0.02	0.986	8731034.54 (0.00 ~ Inf)
Gastric tube					
0					1.00 (Reference)
1	1.14	0.36	3.19	0.001	3.12 (1.55 ~ 6.28)
Urinary tube					
0					1.00 (Reference)
1	1.85	0.37	4.94	<.001	6.37 (3.06 ~ 13.26)
Central venous catheter					
0					1.00 (Reference)
1	2.13	0.40	5.36	<.001	8.39 (3.85 ~ 18.26)
Drainage tube					
0					1.00 (Reference)
1	0.63	0.63	1.00	0.318	1.87 (0.55 ~ 6.40)
Total Parenteral Nutrition					
0					1.00 (Reference)
1	17.01	1029.12	0.02	0.987	24326759.33 (0.00 ~ Inf)
Invasive mechanical ventilation					
0					1.00 (Reference)
1	4.09	1.06	3.85	<.001	59.61 (7.43 ~ 478.04)
Sedation					
0					1.00 (Reference)
1	3.51	1.09	3.24	0.001	33.60 (4.01 ~ 281.81)
Anticoagulant					
0					1.00 (Reference)
1	0.50	0.35	1.40	0.160	1.64 (0.82 ~ 3.28)
Vasoactive drug					
0					1.00 (Reference)
1	3.03	0.60	5.02	<.001	20.62 (6.33 ~ 67.20)
Glucocorticoid					
0					1.00 (Reference)
1	1.38	0.36	3.84	<.001	3.98 (1.96 ~ 8.06)
Age	0.01	0.01	0.43	0.664	1.01 (0.98 ~ 1.03)
BMI	0.06	0.04	1.41	0.158	1.06 (0.98 ~ 1.15)
Temperature	0.88	0.25	3.45	<.001	2.40 (1.46 ~ 3.95)
Heartbeat	0.05	0.01	3.62	<.001	1.05 (1.02 ~ 1.07)
Respiratory rate	0.08	0.10	0.85	0.396	1.09 (0.90 ~ 1.31)
Systolic pressure	0.01	0.01	1.30	0.193	1.01 (0.99 ~ 1.03)
Diastolic pressure	0.04	0.01	3.06	0.002	1.04 (1.02 ~ 1.07)
MAP	0.04	0.01	2.62	0.009	1.04 (1.01 ~ 1.06)
PH	-3.52	2.55	-1.38	0.168	0.03 (0.00 ~ 4.39)
PaO_2_	0.01	0.01	1.76	0.079	1.01 (1.00 ~ 1.02)
PaCo_2_	0.01	0.02	0.41	0.683	1.01 (0.96 ~ 1.06)
Oxygenation index	0.01	0.00	2.40	0.016	1.01 (1.01 ~ 1.01)
HCO^3-^	-0.03	0.05	-0.54	0.586	0.97 (0.88 ~ 1.07)
Lac	0.16	0.11	1.42	0.156	1.17 (0.94 ~ 1.47)
WBC	-0.01	0.04	-0.22	0.827	0.99 (0.92 ~ 1.07)
N	1.06	1.23	0.86	0.390	2.88 (0.26 ~ 31.93)
L	-3.46	1.90	-1.83	0.068	0.03 (0.00 ~ 1.29)
Hb	-0.01	0.01	-0.76	0.450	0.99 (0.98 ~ 1.01)
PLT	-0.00	0.00	-1.62	0.105	1.00 (0.99 ~ 1.00)
CRP	0.03	0.03	1.03	0.305	1.03 (0.98 ~ 1.08)
IL-6	0.00	0.00	0.49	0.621	1.00 (1.00 ~ 1.00)
PCT	0.03	0.04	0.76	0.445	1.03 (0.95 ~ 1.11)
Album	-0.04	0.03	-1.28	0.201	0.96 (0.89 ~ 1.02)
Total albumin	-0.03	0.02	-1.38	0.167	0.97 (0.93 ~ 1.01)
ALT	0.00	0.00	1.49	0.137	1.00 (1.00 ~ 1.01)
AST	0.00	0.00	0.75	0.456	1.00 (1.00 ~ 1.01)
Total bilirubin	0.03	0.02	1.70	0.090	1.03 (1.00 ~ 1.07)
Direct bilirubin	0.06	0.03	1.77	0.076	1.06 (0.99 ~ 1.12)
Urea	0.10	0.03	2.99	0.003	1.10 (1.03 ~ 1.18)
Creatinine	0.00	0.00	1.54	0.124	1.00 (1.00 ~ 1.01)
K^+^	0.26	0.32	0.80	0.423	1.30 (0.69 ~ 2.45)
Na^+^	0.03	0.03	0.97	0.331	1.03 (0.97 ~ 1.08)
Cl^+^	-0.00	0.03	-0.01	0.988	1.00 (0.95 ~ 1.05)
PT	0.03	0.05	0.60	0.548	1.03 (0.93 ~ 1.14)
APTT	0.02	0.01	1.44	0.151	1.02 (0.99 ~ 1.05)
PTA	-0.01	0.01	-0.58	0.559	0.99 (0.98 ~ 1.01)
Fib	-0.01	0.11	-0.12	0.903	0.99 (0.79 ~ 1.23)
D-D	0.07	0.06	1.07	0.286	1.07 (0.95 ~ 1.20)

**Figure 2:**
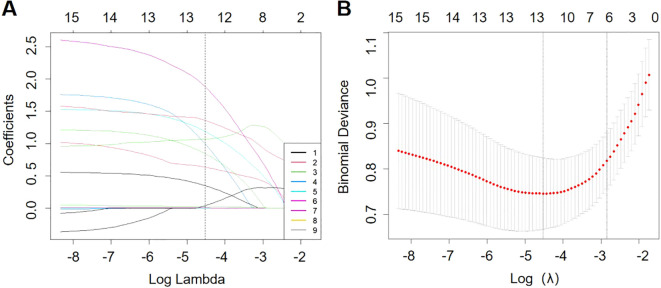
Overview of LASSO coefficients for 15 risk factors **(A)**. Six risk factors were selected using LASSO regression analysis **(B)**.

The use of LASSO regression analysis identified six possible predictors that were found to be significantly associated with pneumonia-associated bloodstream infections (PABSIs) when the λ value was set to 1 standard error (1-se). The identified predictors were long-term antibiotic use, urinary catheter use, central venous catheterization, invasive mechanical ventilation, vasodilator use and glucocorticoid use. For further details, please refer to **Figure 2**.

Further multivariate logistic regression analysis was used to verify the six potential predictors selected by LASSO regression. The results of the analysis showed that the following factors were significantly associated with the risk of pneumonia-associated bloodstream infections (PABSIs): invasive mechanical ventilation, glucocorticoid use, use of long-term antibiotics, use of vasoactive drugs, urinary catheter use, and central venous catheter use. See **Table 3** for details.

**Table 3:** Multivariate logistic regression.

Variables	β	S. E	Z	*P*	OR (95%CI)
Intercept	-2.76	0.36	-7.67	<.001	0.06 (0.03 ~ 0.13)
Urinary tube					
0					1.00 (Reference)
1	0.88	0.54	1.62	0.106	2.40 (0.83 ~ 6.96)
Invasive mechanical ventilation					
0					1.00 (Reference)
1	2.54	1.17	2.18	0.030	12.71 (1.29 ~ 125.54)
Glucocorticoid					
0					1.00 (Reference)
1	1.28	0.43	2.96	0.003	3.61 (1.54 ~ 8.43)
Long-term antibiotic use					
0					1.00 (Reference)
1	1.89	1.05	1.79	0.073	6.62 (0.84 ~ 52.12)
Vasoactive drug					
0					1.00 (Reference)
1	1.53	0.92	1.66	0.096	4.61 (0.76 ~ 27.93)
Central venous catheter					
0					1.00 (Reference)
1	0.21	0.73	0.29	0.775	1.23 (0.30 ~ 5.13)

### 3.3 Construction of a prediction model

**Figure 3:**
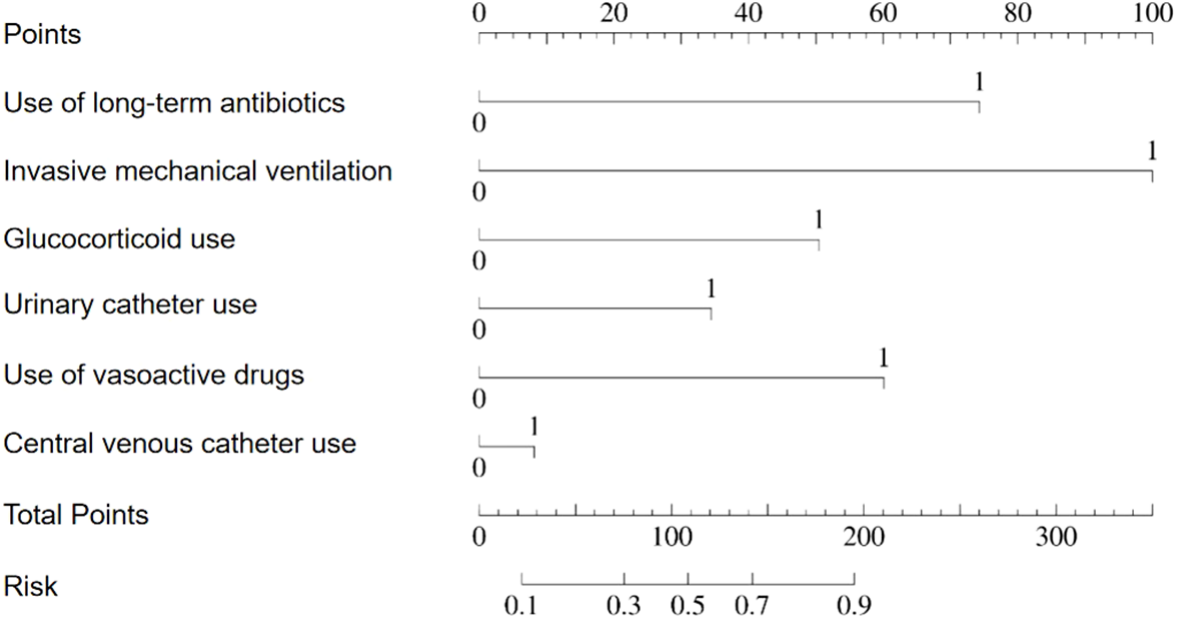
Nomogram for predicting the risk probability of pneumonia-associated bloodstream infections.

Based on the results of the multifactor logistic regression analysis, a prediction model was constructed to predict the risk of pneumonia-associated bloodstream infections. The model is presented visually in a line graph for ease of clinical application and interpretation. For further details, please refer to **Figure 3**.

### 3.4 Model evaluation

To comprehensively evaluate the performance of the nomogram we constructed, we used a number of statistical validation methods. These included receiver operating characteristic (ROC) curve analysis, area under the curve (AUC) calculation, calibration curve analysis and decision curve analysis (DCA). Together, these methods provide insight into the predictive accuracy of the nomogram, its calibration and its use in clinical decision making.

#### 3.4.1 ROC curve and AUC analysis

**Figure 4:**
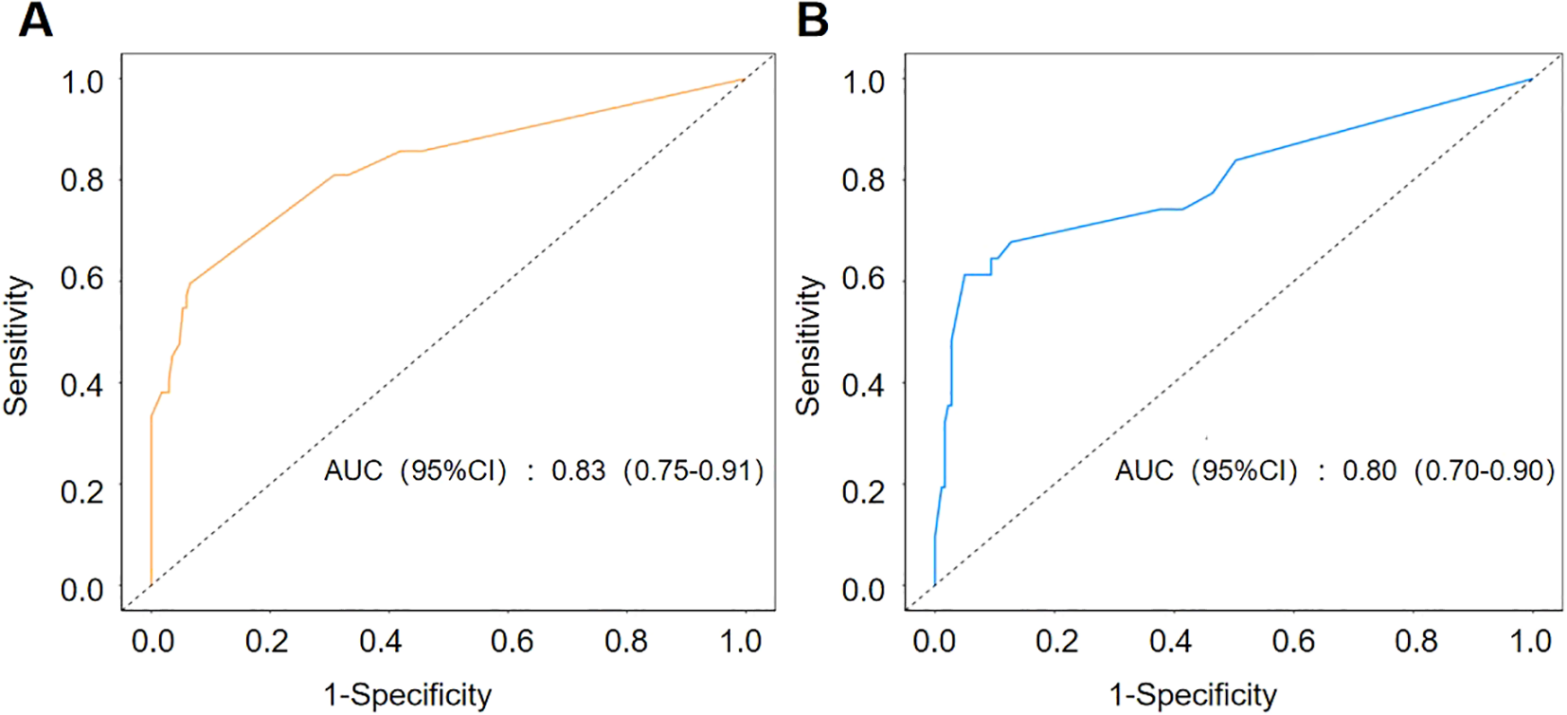
ROC curve of nomogram division with binomial verification of the training group **(A)** and test group **(B)**. The AUC of the training group and the test group was 0.83 and 0.80, respectively.

In order to evaluate the discriminatory power of the nomogram that we had constructed, we initially employed a receiver operating characteristic (ROC) curve analysis. The area under the curve (AUC) values of the nomogram in the training and validation sets reached 0.83 and 0.80 (see **Figure 4**), respectively. These results demonstrate that the model exhibits excellent discriminative power on both the training and validation sets. The high AUC values also indicate that the model has high accuracy in identifying pneumonia-associated bloodstream infections (PABSIs).

#### 3.4.2 Calibration curve

**Figure 5:**
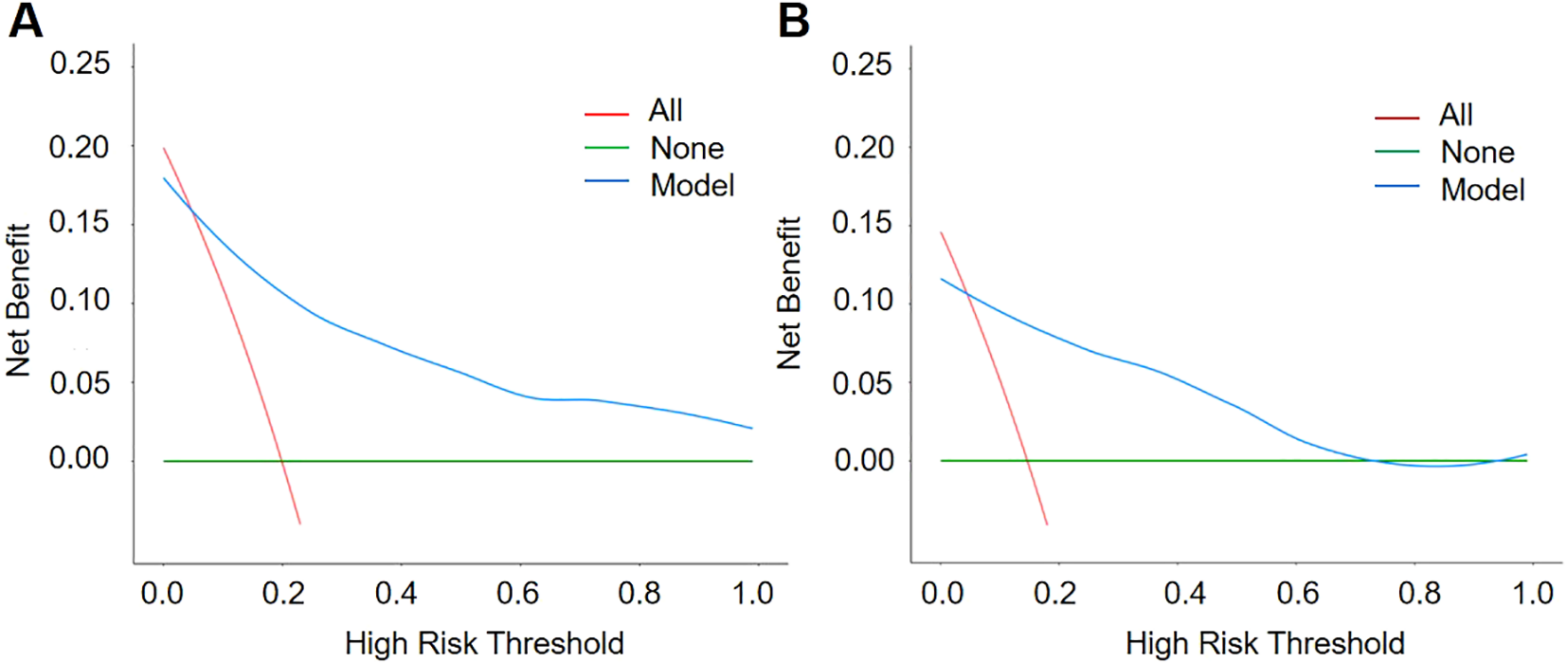
Calibration curve verifies the calibration of nomogram.

In order to ascertain the predictive accuracy of the nomogram, a calibration curve analysis was conducted. The results are presented in **Figure 5**, which demonstrate that the nomogram exhibits robust predictive performance on both the training and validation sets. The calibration curve is in close proximity to the diagonal, indicating that the probability predicted by the model is highly consistent with the actual observed probability of event occurrence. These findings provide further confirmation of the model’s predictive accuracy.

#### 3.4.3 Decision curve analysis

**Figure 6:**
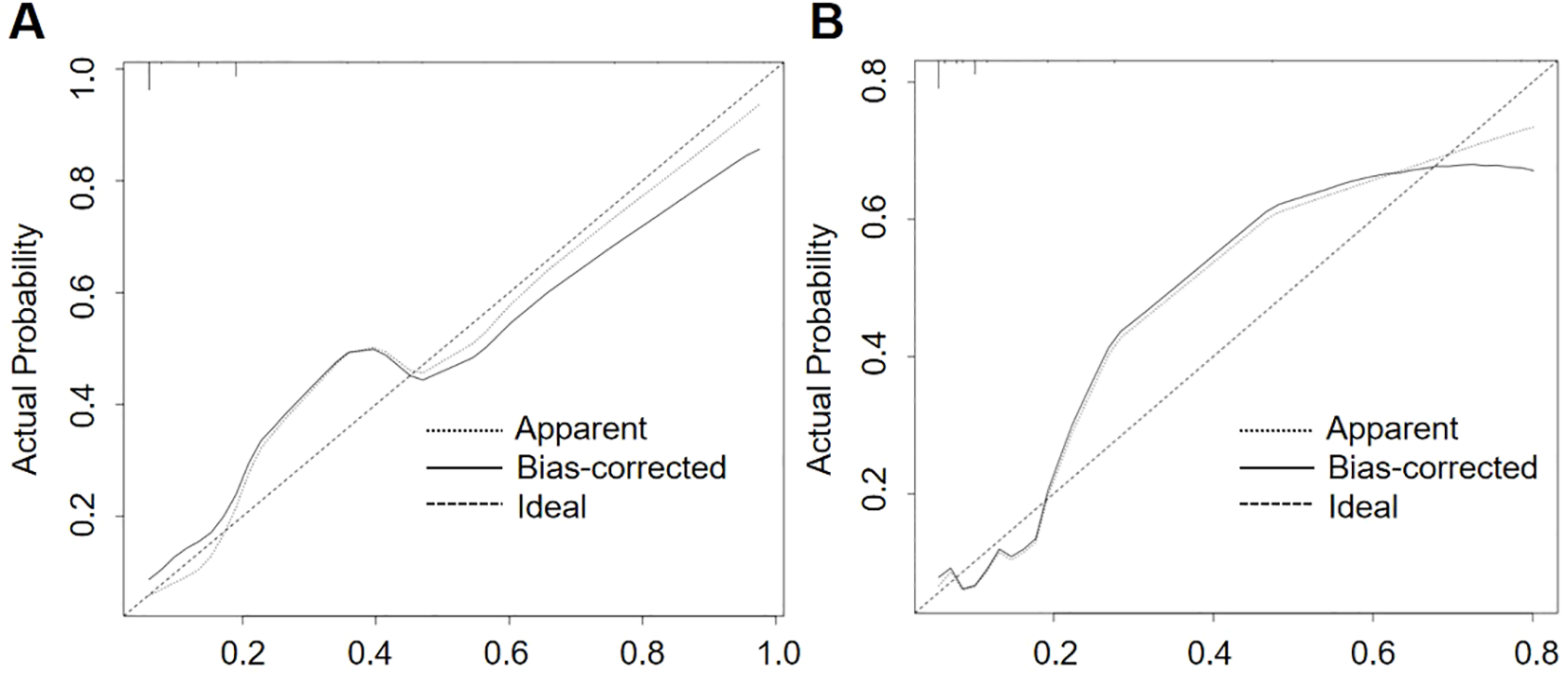
Decision Curve Analysis (DCA) curves of the training group **(A)** and the test group **(B)**. The net benefit of the nomogram was verified by the DCA curves of the two cohorts.

Decision curve analysis (DCA) further verified the value of the nomogram in clinical applications, as shown in **Figure 6**. The results of the DCA showed that the model could achieve above average net benefits regardless of the threshold probability setting, indicating that the nomogram has significant advantages in clinical decision making.

#### 3.4.4 Cross validation

To further validate the model, 5-fold cross-validation was performed. The AUC scores for each fold were as follows: 0.84, 0.77, 0.71, 0.87, and 0.82, with an average AUC of 0.80 ± 0.05. These results indicate that the model showed stable performance across different subsets of the data, with good generalizability. The cross-validation results further support the robustness and reliability of the predictive model.

## 4 Discussion

This study employed a combination of logistic regression and LASSO regression to develop a clinical prediction model for pneumonia-associated bloodstream infections (PABSIs) with notable success. The model employs clinical laboratory indicators and is trained and evaluated using medical record data. The model is capable of accurately predicting the risk of pneumonia patients developing bloodstream infections. The model demonstrated excellent performance on the training and validation sets, with AUC values of 0.83 and 0.80, respectively, confirming its excellent discrimination and prediction capabilities. Furthermore, the calibration curve and decision curve analysis demonstrate that the model exhibits high accuracy in actual prediction and clinical application, providing a robust foundation for clinical decision-making.

To ensure the adequacy of the sample size, this study performed sample size estimation based on the Peduzzi criteria, ensuring sufficient statistical power for the research. In the total sample, the number of outcome events per predictor variable was 12.17 (73 ÷ 6), which is significantly higher than the recommended minimum of 10 events per variable by the Peduzzi criterion, thereby ensuring the robustness and reliability of the model.

For variable selection, this study employed LASSO regression. By introducing a penalty term, LASSO regression effectively selects and shrinks variables, reducing the risk of over-parameterization and mitigating the overfitting problems commonly encountered with small sample sizes, thereby enhancing the model’s predictive capability.

To further assess the robustness and accuracy of the model, the study used the Hosmer-Lemeshow test and calibration curves. The results indicated that the model exhibited good fit and strong predictive ability in both the training and validation sets. The validation set included 31 patients who developed pneumonia-associated bloodstream infections (PABSI), with an event-to-variable ratio of 5.17:1, which meets the basic requirements for regression analysis. The results from the validation set further reinforced the reliability and stability of the model.

In summary, this study ensured the robustness of the model through LASSO regression, Hosmer-Lemeshow test, and calibration curves, thus effectively assessing the risk of pneumonia-associated bloodstream infections.

The results of the study analysis identified several significant predictors associated with pneumonia-associated bloodstream infections (PABSIs), including long-term antibiotic use, use of urinary and central venous catheters, use of invasive mechanical ventilation, use of vasopressors, use of glucocorticoids, and use of urinary catheters.

In recent years, public health authorities and scientific societies around the world have articulated profound concern regarding the escalating issue of antimicrobial resistance (AMR). Antimicrobial resistance (AMR) has been widely acknowledged as a significant global health challenge of the 21st century (Tabah et al., 2023). In clinical practice, the misuse of antibiotics has been the subject of considerable attention, and their overuse has been identified as a significant contributing factor to the development of drug resistance (Read and Woods, 2014). The long-term use of antibiotics can result in selective changes to the structure of the human microbiota, whereby susceptible strains are suppressed or eliminated, while resistant strains survive and proliferate. This increase in selective pressure has resulted in an increase in the frequency of multidrug-resistant strains (Meng et al., 2021). These resistant strains demonstrate reduced susceptibility to the majority of antimicrobial drugs. As drug resistance increases, the treatment of lung infections becomes more challenging, leading to an increase in the prevalence of pneumonia-associated bloodstream infections. The results of the present study demonstrate a robust correlation between long-term antibiotic utilization and pneumonia-associated bloodstream infections, which is consistent with the findings of prior studies in this area. Such an association may be attributed to a dysbiosis, or disruption, of the patient’s endogenous flora, thereby elevating the risk of bloodstream infections among patients diagnosed with pneumonia. These findings have significant clinical implications, emphasizing the necessity for more precise and efficacious prevention and treatment strategies. The introduction of a defined daily dose (DDD) for each individual patient represents a crucial step in the effective management of those with pneumonia.

Device-associated infections have been shown to remain a significant challenge in clinical practice, primarily due to their high prevalence and potential for mortality (Blot et al., 2005; Li, 2015). In particular, urinary and central venous catheters provide a pathway for bacteria to enter the bloodstream, serving as a common conduit for hospital-acquired infections. In this study, we focused on the cohort of patients with pneumonia and found a notable association between urinary and central venous catheters and pneumonia-associated bloodstream infections after excluding other potential sources of bloodstream infections. This association may be due to the fact that patients with these catheters tend to have poorer overall health, higher disease severity and poorer prognosis. In addition, these patients often require prolonged periods of bed rest, which can affect their hemodynamic status.

Invasive mechanical ventilation is a fundamental aspect of respiratory and critical care medicine. It allows patients with respiratory distress to maintain adequate oxygenation and carbon dioxide elimination, thereby improving oxygenation and reducing the incidence of hypoxemia. In patients with fatigued or weak respiratory muscles, invasive mechanical ventilation can reduce the burden on the respiratory muscles and provide the necessary respiratory support for other treatments. However, the establishment of an artificial airway also deprives the patient of the airway’s natural defense mechanisms, such as the ability to heat, humidify, and clear the inspired air. This increases the risk of airborne pathogens entering the patient’s airways and lungs. In addition, prolonged use may result in tracheal damage (Eggimann and Pittet, 2001). Current research suggests that the presence of an endotracheal tube is the most significant risk factor for the development of ventilator-associated pneumonia (VAP) (Walaszek et al., 2016). The present study also identified invasive mechanical ventilation as a risk factor for the progression of pneumonia patients to pneumonia-associated bloodstream infections. Because the establishment of an artificial airway creates a direct pathway between the lungs and the outside world, increasing the possibility of invasion by pathogenic microorganisms from the outside world, it may further exacerbate pneumonia and thus increase the risk of bloodstream infections.

Vasopressors are of great importance in critical care medicine, particularly in the context of resuscitation and treatment of shock. These drugs can significantly improve the patient’s hemodynamics, control blood pressure, and optimize blood flow to vital organs (Annane et al., 2018). In patients with pneumonia, pathogens of pulmonary infection, including bacteria, viruses or fungi, can enter the bloodstream through various mechanisms and cause bloodstream infections. During infection, pathogens such as viruses or bacteria can directly infect epithelial cells, causing cell death (Kenney et al., 2017). Tissue damage and inflammatory responses can lead to disruption of the alveolar-capillary barrier, allowing pathogens to cross the damaged lung tissue and enter the bloodstream (Long et al., 2022). In addition, the inflammatory response in the infected area may lead to local vasodilation and increased vascular permeability, facilitating pathogen invasion. Once the pathogen enters the blood stream, it can cause a systemic infection, which is a bloodstream infection. Studies have shown that the use of vasoactive drugs is significantly associated with the incidence of pneumonia-associated bloodstream infections. This may facilitate the occurrence of bloodstream infections by affecting the vasodilator and permeability properties of blood vessels due to damage to the alveolar-capillary barrier.

The pro-inflammatory mediator storm that critically ill patients with pulmonary infections may experience represents a complex pathological process in which an imbalance between pro- and anti-inflammatory mediators can lead to a variety of adverse effects (Li et al., 2020). Glucocorticoids help to attenuate the inflammatory response in critically ill patients by inhibiting the release of pro-inflammatory mediators, thereby regulating excessive immune responses and improving clinical symptoms (Patel et al., 2020). However, this treatment strategy may also suppress the patient’s immune function, thereby reducing their ability to resist new infections. This may provide opportunities for invasion and spread of pathogenic microorganisms, and may even lead to immunosuppression and uncontrolled inflammatory responses. The results of this study demonstrate an increased likelihood of pneumonia-associated bloodstream infections in patients treated with glucocorticoids. This may be due to the immunosuppressive effects of glucocorticoids, which may increase the likelihood of such infections.

The particular advantage of this model over existing studies is that it combines the advantages of LASSO regression in feature selection with the strengths of logistic regression in predictive ability, thereby enhancing the stability and predictive accuracy of the model. This is evidenced by the high AUC and exemplary calibration curve performance of this model. In addition, this study focuses on the risk of pneumonia patients developing bloodstream infections, which is a critical aspect of pneumonia patient management. Given the current lack of research on individuals with pneumonia-associated bloodstream infections, this study seeks to assess the risk of such infections by developing a predictive model to facilitate the identification of early-stage pneumonia patients who may subsequently develop bloodstream infections. This will facilitate the implementation of prompt treatment measures and contribute to a reduction in mortality rates.

Although this model has demonstrated considerable efficacy in predicting pneumonia-associated bloodstream infections, it is important to acknowledge the limitations inherent in its evaluation. First, because this study used a retrospective cohort study design, there is a possibility of selection and information bias. Second, the study sample was derived from a single center, which may limit the generalizability of the model. Therefore, it is recommended that future studies use a multicenter prospective study design to further validate and optimize this model. Furthermore, future studies could explore the potential of combining this model with other biomarkers or imaging features to improve the accuracy of prediction and the value of clinical application. At the same time, external validation of the model and its applicability in different populations are important directions for future research.

## 5 Conclusion

In conclusion, the clinical prediction model for pneumonia-related bloodstream infections developed in this study, based on LASSO regression and logistic regression, provides clinicians with a precise risk assessment tool specifically for the management of pneumonia patients. The model not only has high predictive accuracy, but also has good calibration performance, which allows clinicians to identify high-risk patient groups early. The implementation of timely and effective preventive and treatment measures based on this tool is expected to significantly improve the clinical prognosis of patients.

## Data Availability

Ethical approval has been signed, and the data from this research project will be kept only by the researcher, will not be uploaded to public databases, and will not be shared with other researchers. Requests to access the datasets should be directed to ZZ, purple_zzt@163.com.
